# Slow recruitment in the HIMALAIA study: lessons for future clinical trials in patients with delayed cerebral ischemia after aneurysmal subarachnoid hemorrhage based on feasibility data

**DOI:** 10.1186/s40814-022-01155-4

**Published:** 2022-08-30

**Authors:** Celine S. Gathier, Mathieu van der Jagt, Walter M. van den Bergh, Jan Willem Dankbaar, Gabriel J. E. Rinkel, Arjen J. C. Slooter, for the HIMALAIA Study Group, Ale Algra, Jan-Willem Dankbaar, Celine S. Gathier, Jozef Kesecioglu, Gabriel J. E. Rinkel, Irene C. van der Schaaf, Arjen J. C. Slooter, Bon H. Verweij, Ruben Dammers, Diederik W. J. Dippel, Clemens M. F. Dirven, Mathieu van der Jagt, Fop van Kooten, Aad van der Lugt, Walter M. van den Bergh, Bert A. Coert, Marcella C. Müller, W. Peter Vandertop, Guus N. Beute, Annemarie W. Oldenbeuving, Bram van der Pol, Gerwin Roks, Willem Jan J. van Rooij, Menno Sluzewski

**Affiliations:** 1 Department of Intensive Care Medicine, UMC Utrecht Brain Center, University Medical Center Utrecht, Utrecht University, Utrecht, The Netherlands; 2 Department of Neurology and Neurosurgery, UMC Utrecht Brain Center, University Medical Center Utrecht, Utrecht University, 3508 Utrecht, The Netherlands; 3 Department of Intensive Care Adults and Erasmus MC Stroke Center, Erasmus MC — University Medical Center Rotterdam, Rotterdam, The Netherlands; 4 Department of Critical Care, University Medical Center Groningen, University of Groningen, Groningen, The Netherlands; 5 Department of Radiology, UMC Utrecht Brain Center, University Medical Center Utrecht, Utrecht University, Utrecht, The Netherlands; 6 University Medical Center Utrecht, Utrecht University Utrecht, Utrecht, The Netherlands

**Keywords:** Aneurysmal subarachnoid hemorrhage, Delayed cerebral ischemia, Randomized trial

## Abstract

**Background:**

Our randomized clinical trial on induced hypertension in patients with delayed cerebral ischemia (DCI) after aneurysmal subarachnoid hemorrhage (aSAH) was halted prematurely due to unexpected slow recruitment rates. This raised new questions regarding recruitment feasibility. As our trial can therefore be seen as a feasibility trial, we assessed the reasons for the slow recruitment, aiming to facilitate the design of future randomized trials in aSAH patients with DCI or other critically ill patient categories.

**Methods:**

Efficiency of recruitment and factors influencing recruitment were evaluated, based on the patient flow in the two centers that admitted most patients during the study period. We collected numbers of patients who were screened for eligibility, provided informed consent, and developed DCI and who eventually were randomized.

**Results:**

Of the 862 aSAH patients admitted in the two centers during the course of the trial, 479 (56%) were eligible for trial participation of whom 404 (84%) were asked for informed consent. Of these, 188 (47%) provided informed consent, of whom 50 (27%) developed DCI. Of these 50 patients, 12 (24%) could not be randomized due to a logistic problem or a contraindication for induced hypertension emerging at the time of randomization, and four (8%) were missed for randomization. Eventually, 34 patients were randomized and received intervention or control treatment.

**Conclusions:**

Enrolling patients in a randomized trial on a treatment strategy for DCI proved unfeasible: only 1 out of 25 admitted and 1 out of 14 eligible patients could eventually be randomized. These rates, caused by a large proportion of ineligible patients, a small proportion of patients providing informed consent, and a large proportion of patients with contraindications for treatment, can be used to make sample size calculations for future randomized trials in DCI or otherwise critically ill patients. Facilitating informed consent through improved provision of information on risks, possible benefits, and study procedures may result in improved enrolment.

**Trial registration:**

The original trial was prospectively registered with ClinicalTrials.gov (NCT01613235), date of registration 07-06-2012.

## Key messages regarding feasibility

- What uncertainties existed regarding the feasibility?

This article describes the course of a randomized trial in critically ill patients. We performed a feasibility pilot study before commencing with the main trial, mainly focusing on recruitment feasibility and safety. The outcome of the feasibility study was that we would be able to randomize 1 to 2 patients per month per center without major safety issues. With four centers participating, we calculated that we needed 4 to 5 years to enroll 240 patients and to complete the study. As this was deemed a reasonable time period, we pursued with the main study. However, not long after commencing with the main multicenter trial, recruitment turned out to be much slower than expected, which was unforeseen and surprising. Therefore, we became uncertain regarding recruitment success and whether we would be able to finish the main trial.

- What are the key feasibility findings?

Because of the newly risen uncertainty regarding recruitment feasibility, one could see the main trial as a new feasibility phase of the trial. The key finding was that we were unable to recruit sufficient patients, even though several attempts were made to improve recruitment. This lead to preliminary cessation of the trial, as advised by the trial safety monitoring board.

- What are the implications of the feasibility findings for the design of the main study?

We decided to not pursue with another trial. We did, however, assess the reasons for recruitment failure in our study. We found that the large proportion of ineligible patients and the small proportion of patients providing informed consent were the most important reasons for recruitment failure. These findings can be used to optimize sample size calculations when designing future randomized trials on treatment strategies for DCI or trials seeking to include critically ill patients. Furthermore, optimizing the informed consent procedure may result in improved enrolment of these patients.

## Background

Delayed cerebral ischemia (DCI) occurs in around 30% of aneurysmal subarachnoid hemorrhage (aSAH) patients between days 3 and 14 after the initial hemorrhage and is an important contributor to poor outcome [1]. Although several treatment strategies for DCI have been investigated [2], only one randomized trial has been published on a strategy to treat DCI [3], resulting in paucity of evidence to direct treatment in these patients.

One of the reasons for the lack of trials in this condition may be that obtaining informed consent from patients at the time they develop DCI can be challenging. Patients with DCI often cannot provide informed consent themselves because of an impaired level of consciousness, and thus, consent should be obtained from proxies. Obtaining timely informed consent from proxies is also challenging as the investigated treatment has to be installed as soon as possible after the onset of symptoms. Therefore, we developed another approach of asking informed consent and performing randomization in our monocenter pilot randomized trial in patients with DCI, in which we investigated the effectiveness of induced hypertension on outcome at 3 months (primary outcome) and cerebral perfusion assessed with CT perfusion (secondary outcome measure). All eligible patients, or their legal representatives in case of a depressed level of consciousness, were asked for informed consent as soon as possible after admission. Randomization was only performed at time of development of DCI. As this resulted in adequate inclusion rates during the pilot trial, we continued with the main trial (the HIMALAIA study [3, 4]).

However, not long after commencing with the main multicenter trial, recruitment turned out to be much slower than expected, which was unforeseen and unexpected. It became uncertain whether it would be feasible to continue with the main trial, especially after several attempts to improve recruitment failed. Eventually, the trial was halted prematurely due to insufficient recruitment. Regarding recruitment, our trial can be seen as a feasibility trial of which important lessons can be learned. Therefore, we aimed to explore the reasons for the slower than expected recruitment to facilitate the design of future randomized trials in aSAH patients with DCI or other trials seeking to include critically ill patients.

## Methods

From February 2009 until January 2015, the HIMALAIA Study was performed in four centers in the Netherlands [3, 4]. The in- and exclusion criteria for trial participation are shown in Table 1. A detailed description of the trial methods have been published elsewhere [3, 4]. From the four centers, two (the Amsterdam University Medical Center, location Academic Medical Center (AMC, 5 included patients), and the Elisabeth-TweeSteden Hospital, Tilburg (ETZ, 2 included patients)) were not included in the present study because the trial could not run its optimal course in both centers. The AMC stopped including patients after 2 years because of the desire to initiate another intervention trial in aSAH patients, which could not be performed simultaneously with the HIMALAIA study as judged by the medical ethics board of the AMC. The ETZ was added lastly as a trial site and thus had only been including patients for 2 years when the trial was halted. Therefore, as these two centers only included patients for 2 years and the reasons for no longer including patients were not influenceable by the HIMALAIA investigators, we felt that the course in these 2 centers was not representative for the main course of the trial.

**Table 1:** Criteria for eligibility and randomization

**Criteria for eligibility**	
**Inclusion criteria**	
Age above 18 years	
SAH with an aneurysmal bleeding pattern	
**Exclusion criteria**	
Evidence of DCI after the SAH, unless symptoms of DCI started within 3 h	
Coexisting severe head injury	
Perimesencephalic hemorrhage	
A history of a ventricular cardiac rhythm disorder or heart failure necessitating medical treatment	
Likely transfer to another hospital, not participating in the trial, soon after treatment for the aneurysm	
Moribund	
Pregnancy	
No informed consent or informed consent not feasible	
*Additional exclusion criteria for the sub-study on cerebral perfusion*	
Known allergy for CT contrast agents	
Renal failure, defined as a serum creatinine > 150 μmol/L	
Diabetes mellitus and glomerular filtration rate < 60	
**Criteria for randomisation**	
**Inclusion criteria**	
DCI based on a decrease of at least 1 point on the Glasgow Coma Scale sum score and/or the development of new focal neurological deficits according to the NIHSS, diagnosed by a neurologist, neurosurgeon, intensivist, unless the deterioration does not reflect DCI as evaluated by the treating physician	
**Exclusion criteria**	
Another cause for the neurological deterioration, e.g.	
Increasing hydrocephalus	
Recurrent bleeding	
Clinical signs of epilepsy	
Severe infectious disease with associated decrease in level of consciousness	
Hypoglycemia, defined as serum glucose < 3.0 mmol/L	
Hyponatremia, defined as serum sodium < 125 mmol/L	
Metabolic encephalopathy due to renal or hepatic failure	
Ischemia related to aneurysm treatment	
An untreated symptomatic aneurysm	
A spontaneous mean arterial pressure above 120 mmHg at the moment of randomization	
Any other contraindication for induced hypertension	
No CT-perfusion scan at time of neurological deterioration	
More than three contrast CT scans since admission	

*CT* Computerized tomography, *NIHSS* National Institutes of Health Stroke Scale, *SAH* Subarachnoid hemorrhage, *DCI* Delayed cerebral ischemia

For the current study, we thus assessed the course of the trial in the two centers that screened most patients during a representable period of the course of the trial: the University Medical Center Utrecht, Utrecht (UMCU, 26 included patients), and the Erasmus MC University Medical Center, Rotterdam (EMC, 8 included patients).

In both centers, all aSAH patients were screened for eligibility as soon as possible after hospital admission by the study team. Informed consent was asked from eligible patients or their legal representatives in case the patient had an impaired level of consciousness by the principle investigator, study coordinator, or resident in neurology who was specifically trained for the informed consent procedure of the trial. Informed consent was asked as soon as possible after hospital admission during office hours. The verbal information consisted of information about the rationale of the trial, the randomization procedure, detailed information about the possible effect and side effects of induced hypertension, and the additional burden associated with trial participation (transfer to the intensive care unit (ICU) in case of randomization to induced hypertension, an additional computerized tomography-perfusion scan (CT perfusion) in case of participation in the CT-perfusion sub-study and a telephone interview at 3 and 12 months after hospital admission). Besides verbal information, the patient or his/her representative was also given written information about the trial. This consisted of a 4-page information document containing all information that was also provided verbally. Patients or legal representatives were always allowed at least 1 day to consider trial participation, and additional verbal information was provided when asked for. If consent was provided, a notification was made in the patient’s medical file, and all involved medical personnel was informed. Randomization was performed only in patients who developed DCI, as soon as possible after diagnosis of DCI. DCI was defined as a decrease of at least 1 point on the Glasgow Coma Scale sum score or development of new focal neurological deficits at least 1 h, or both, with exclusion of other prespecified explanations for clinical deterioration.

During the course of the trial, four investigators took shifts for the trial telephone, a phone number that could be called 24 h per day for discussing randomization of potential candidates. The medical personnel was instructed to call the trial number as soon as a patient in whom informed consent was obtained developed signs of clinical deterioration and to order a CT scan of the brain and blood examination according to the trial protocol. In the meantime, the trial investigator checked for the availability of an ICU bed, if the patient was not yet in the ICU, to be able to facilitate induced hypertension. As soon as other causes for the clinical deterioration had been ruled out, an ICU bed was available, and no contraindications for induced hypertension had developed after informed consent was provided; randomization was performed by the investigator using a computer randomization program. A detailed description of the interventions per group have been published elsewhere [3, 4].

From the prospectively collected screening and enrollment logs and additional review of the medical files, we extracted the following: (1) how many aSAH patients were admitted within the risk period for development of DCI, defined as admission < 14 days after ictus, (2) how many were screened for eligibility and how many eligible patients were contacted, (3) how many provided informed consent, (4) how many of these patients developed DCI, and (5) how many could eventually be randomized. Apart from the patients who participated in the trial, we assessed from the medical files whether DCI developed in patients who did not participate in the trial. The medical ethics committee of the UMCU and EMC waived the need for informed consent for the current study on recruitment (protocol numbers 15-486/C and MEC-2012-170, respectively).

## Results

**Fig. 1:**
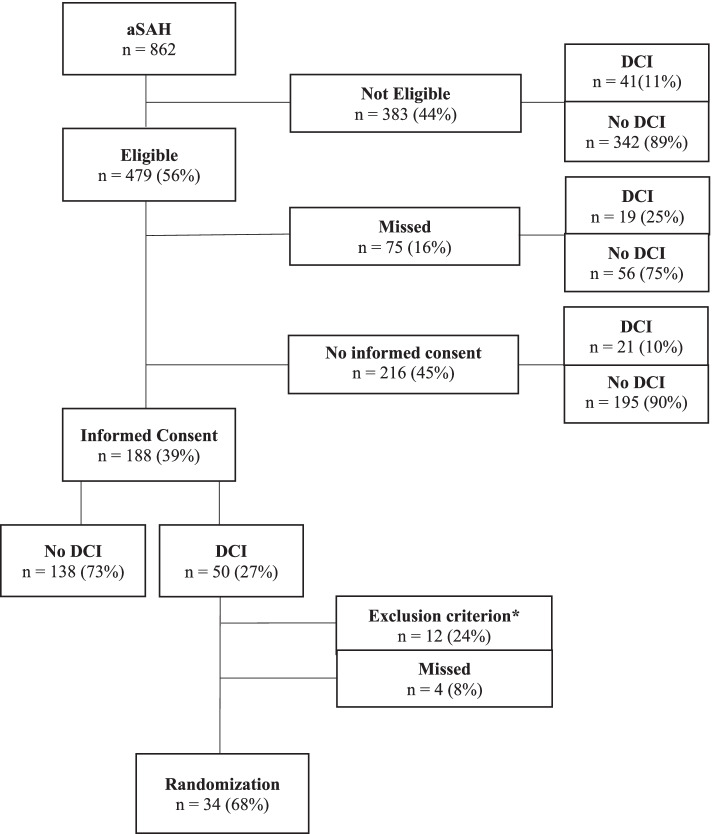
Flowchart of inclusion of patients aSAH: aneurysmal subarachnoid hemorrhage. DCI: delayed cerebral ischemia. * Blood pressure already too high (*n *= 5), spontaneous improvement (*n *= 3), initial doubt whether the deterioration was based on DCI (*n *= 2), DCI developed just after transferal to another hospital (*n *= 1), no ICU bed available (*n *= 1). For detailed description of exclusion criteria, see text in Methods section

From February 2009 until January 2015, 862 aSAH patients were admitted to the UMCU (*n* = 672) or EMC (*n* = 190). Figure 1 shows in detail how patients were retrieved for the trial. Of the 862 aSAH patients admitted during the course of the trial, 479 (56%) were eligible for trial participation. Patient characteristics are shown in Table 2. The reasons for non-eligibility are provided in Table 3. The most frequent reasons were moribund status, the absence of a symptomatic aneurysm, or the impossibility to (entirely) treat the aneurysm. Sixteen patients (1.9% of all screened patients) were incorrectly deemed ineligible. Of the 479 eligible patients, 75 (16%) were not approached (missed) for informed consent, of whom 19 developed DCI and thus could potentially have been candidates for trial participation after informed consent. Of the 404 (84%) patients who were approached for informed consent, 188 (47%) provided it, of whom 50 (27%) developed DCI (Fig. 1). Of these 50 patients, 34 (68%) could be randomized (26 in the UMCU and 8 in the EMC) and managed according to intervention or control treatment. In 12 (24%) patients, randomization could not be performed: in five patients, baseline blood pressure was too high; in three patients, the deterioration rapidly improved spontaneously; and in two patients, there was initial doubt whether the deterioration was based on DCI: one patient developed DCI just after transfer to another hospital, and in one patient, no ICU bed was available. Four (8%) patients were not randomized despite the absence of contraindications for induced hypertension.

**Table 2:** Patient characteristics

	All patients (***n*** = 862)	Eligible (***n*** = 479)	Not eligible (***n*** = 383)
UMCU/EMC (%)	672 (78%)/190 (22%)	373 (78%)/106 (22%)	299 (78%)/84 (22%)
Age, mean (SD)	59 (13)	57 (12)	61 (14)
Female (%)	603 (70%)	359 (75%)	244 (64%)
Medical history of hypertension (%)	256 (30%)^a^	122 (26%)^b^	134 (35%)^c^
Admission-WFNS score > 3 (%)	373 (43%)	148 (30%)	225 (59%)
Anterior circulation aneurysm (%)	506 (59%)	328 (68%)	178 (46%)
No aneurysm with aneurysmal bleeding pattern (%)	97 (11%)	-	97 (25%)
Aneurysm treatment (%)			
Clip	254 (30%)	208 (43%)	49 (13%)
Coil	365 (42%)	271 (57%)	93 (24%)
No aneurysm treatment	243 (28%)	-	241 (63%)

*UMCU* University Medical Center Utrecht, *EMC* Erasmus MC University Medical Center, *SD* Standard deviation, *WFSN* World Federation of Neurosurgical Societies scale, *DCI* Delayed cerebral ischemia. ^a^Missing for 10 patients. ^b^Missing for 1 patient. ^c^Missing for 9 patients

**Table 3:** Reasons for non-eligibility for the trial

	Number of patients ***n*** = 383
**Reasons for non-eligibility (%)**	
Contraindication for participation	367 (96%)
Moribund on admission	160 (44%)^a^
No aneurysm found	67 (18%)
Aneurysm could not (entirely) be treated	47 (13%)
Partial coiling	9
Delayed aneurysm treatment due to severe vasospasm	11
Aneurysm treatment technically not possible	27
Signs of DCI on admission	23 (6%)
Cardiac contraindication for iHT	18 (5%)
Transfer to another hospital	17 (5%)
Contraindication for CT-contrast agent	10 (3%)
Noncardiac contraindication for iHT	7 (2%)
No Dutch or English	6 (2%)
Legally incompetent patient without a legal representative present	5 (1%)
DCI immediately after treatment of the aneurysm	3 (1%)
Admission after DCI risk period^b^	1 (0.3%)
Pregnancy	1 (0.3%)
Treatment restriction requested by family	1 (0.3%)
Initially not recognized aSAH	1 (0.3%)
Incorrectly deemed not eligible	16 (4%)
Delayed admission to the hospital without any signs of DCI yet	5 (31%)
Not asked for informed consent due to misinterpretation of the in- and exclusion criteria for eligibility while they were actually eligible	11 (69%)

^a^Three patients eventually did not die during admission. ^b^Admission after the risk period for DCI had already passed, defined as admission after 14 days after the initial hemorrhage. *DCI* Delayed cerebral ischemia, *iHT* Induced hypertension, *aSAH* Aneurysmal subarachnoid hemorrhage

## Discussion

We found several factors explaining the slower than expected recruitment. Almost half of the patients admitted during the study period were not eligible for the trial; of those who were eligible, more than half declined participation in the trial, and of those who developed DCI, one-third could not be randomized due to emerging contraindications for induced hypertension or a logistical problem.

A substantial proportion of patients was ineligible. However, no unexpected reasons were seen for ineligibility. The small number of patients incorrectly deemed ineligible will not have had major impact on the slow inclusion rate.

Only half of eligible patients provided informed consent, which was a smaller proportion than anticipated. In other randomized trials in which either the informed consent procedure was complex [5], or subjects were critically ill and admitted to the ICU [6–8], the proportion of eligible patients providing informed consent ranged from 75 to 82%. In our trial, patients or their legal representatives were asked for informed consent *in case* DCI would occur. This procedure was chosen to be able to initiate induced hypertension as early as possible after onset of DCI symptoms. However, it may very well be that the complexity of our informed consent procedure was an important reason for the small proportion of patients providing informed consent. Patients or legal representatives may find it difficult to imagine whether they want (their family member) to be treated with induced hypertension or not, in a possible future setting of clinical deterioration. We have no empirical data to support or refute this hypothesis. We also did not systematically assess whether the proportion of obtained informed consent differed when asked by a member of the study team (the principle investigator or study coordinator) as opposed to a resident in neurology. However, we aimed to minimize this difference by individually training the residents beforehand. Due to the fact that we did not systematically assess the reasons for declining informed consent, our study cannot provide solid recommendations. We suggest to systematically assess factors that might influence recruitment as a secondary outcome in a future clinical trial in DCI patients.

A substantial proportion of the patients who provided informed consent and developed DCI could eventually not be randomized due to the presence of either a contraindication for induced hypertension, a logistical boundary such as unavailability of an ICU bed, or because they were not asked (missed) for randomization. Under ideal circumstances, the number of additional patients that could have been randomized is 44 (19 eligible patients who were missed for informed consent and developed DCI, 21 patients who were eligible but did not provide informed consent and developed DCI, and 4 patients who were eligible, provided informed consent, and developed DCI but were missed for randomization). This would have resulted in a total of 78 patients randomized over the course of 6 years (1 out of 11 admitted and 1 out of 6 eligible patients). Also, the numbers of DCI patients that were truly missed either for informed consent (*n* = 19) or for randomization (*n* = 4) were small, and therefore, we feel that this would not have major impact on the inclusion rate.

The overall frequency of DCI in the subset of eligible patients was 19% and in the subset of patients who provided informed consent 27% which was in line with what was expected and used in the sample size calculation for the trial. Therefore, the frequency of DCI itself has probably not influenced the recruitment in our trial.

### Future directions

As we considered our complex informed consent procedure an important contributor to the small proportion of informed consent, other approaches for asking informed consent might be more successful. Various other informed consent procedures are possible, such as the “deferred informed consent” method [9, 10], the “trials within cohorts” method, or the “just-in-time” consent method [11–13]. However, with these methods, all or a proportion of patients are not informed beforehand about the study. Withholding this information when there is actually time to provide it as we have shown in our study is in our view not ethical and not in line with current European guidelines [14]. Therefore, we would still advice the method that we have used for obtaining informed consent.

However, strategies to facilitate decision-making for patients or legal representatives should be explored, such as providing leaflets or short videos that can be viewed by patients and/or relatives in their own time, containing detailed information on DCI, its consequences, and the treatment. Video-assisted informed consent improves patients’ understanding of the proposed treatment or intervention both in clinical practice as in research [15, 16] and may result in faster enrolment with improved enrolment of minorities [17, 18].

Inclusion rates could also be improved by improving the detection of patients with DCI. Especially in patients with a poor clinical condition, development of DCI can be missed when they are monitored by clinical examinations only. Additional diagnostics, such as transcranial Doppler ultrasonography, brain perfusion imaging, and invasive brain multimodality monitoring may improve the timely diagnosis of DCI in these patients [19]. However, as these methods are often expensive and laborious with requirement of specific expertise and devices, they are unpractical in clinical practice and could be a complicating factor for successful implementation of a randomized clinical trial.

Alternative randomized trial study designs could be a controlled, crossover study using cluster randomization, or a stepped-wedge cluster randomized controlled trial.

In a crossover study using cluster randomization, the different treatment regimens (for instance, standard care and the intervention treatment(s)) are assigned to the participating centers in a random order over a specific time course. For instance, one group of clusters receives standard care in the first time period and the intervention in the second, and the other group of clusters receives both treatments in reverse order. In a stepped-wedge cluster randomized controlled trial, all centers (clusters) start with the standard care procedure. After a certain timepoint, the intervention is implemented first in 1 center and thereafter in the other centers in a stepped way, with the order of implementation determined by randomization. After the introduction of the intervention in a certain center, the intervention is continued, so that at the end of the study period, the intervention is performed in all centers (clusters).

However, the success of these specific study designs depends on adherence to the installed regimen per time period with as little as possible dropout (or crossover to another treatment regimen). In the case of our specific trial, we feel that this might have become an obstacle for several reasons. As the use of induced hypertension differed between the participating centers before the start of the trial (for instance, induced hypertension was hardly ever used in the UMCU but more frequently used in the AMC), and as the intervention type (induced hypertension) was high risk, this could have resulted in reluctancy of the involved treating physicians to just simply adhere to an imposed treatment regime for all eligible patients during a specific time period, with the possible result of noncompliance to the protocol. Furthermore, not asking individual participating patients for informed consent, especially when a higher risk intervention is investigated, is in our view, as stated before, unethical and not in line with current European guidelines.

To bypass the difficulties associated with designing and conducting a randomized controlled trial on treatment strategies for DCI patients, a different approach could be to evaluate the effectiveness or safety of an intervention in real-life practice by using data that are obtained during routine clinical care (observational comparative effectiveness research (CER) [20]. Even though data can be collected quickly and easier than in a randomized trial, bias is inevitable which will always prevent providing solid evidence-based recommendations. We would therefore advocate improvement of clinical trials in DCI patients.

## Conclusions

Recruitment in the HIMALAIA trial was mainly hampered by the small proportion of patients providing informed consent and the large proportion of ineligible patients. These are important findings as they can be used for the design and for sample size calculations for future randomized trials in DCI patients or other trials investigating critically ill patients, aiming to increase the possibility of successful completion of such a trial. Improving the informed consent method and detection of DCI in poor grade aSAH patients is important implications for further research, and thus, we urge future researchers to seek multicenter collaboration in trying to find better treatment options for patients with DCI.

## Data Availability

The datasets used and/or analyzed during the current study are available from the corresponding author on reasonable request.

## Abbreviations

AMC: Amsterdam University Medical Center, location Academic Medical Center
aSAH: Aneurysmal subarachnoid hemorrhage
CER: Comparative effectiveness research
CT: Computerized tomography
DCI: Delayed cerebral ischemia
EMC: Erasmus MC University Medical Center
ETZ: Elisabeth-TweeSteden Hospital
ICU: Intensive care unit
iHT: Induced hypertension
NIHSS: National Institutes of Health Stroke Scale
SD: Standard deviation
UMCU: University Medical Center Utrecht
WFNS: World Federation of Neurosurgical Societies scale
