# Correction: Slow recruitment in the HIMALAIA study: lessons for future clinical trials in patients with delayed cerebral ischemia after aneurysmal subarachnoid hemorrhage based on feasibility data

**DOI:** 10.1186/s40814-022-01172-3

**Published:** 2022-09-22

**Authors:** Celine S. Gathier, Mathieu van der Jagt, Walter M. van den Bergh, Jan Willem Dankbaar, Gabriel J. E. Rinkel, Arjen J. C. Slooter, for the HIMALAIA Study Group, Ale Algra, Jan-Willem Dankbaar, Celine S. Gathier, Jozef Kesecioglu, Gabriel J. E. Rinkel, Irene C. van der Schaaf, Arjen J. C. Slooter, Bon H. Verweij, Ruben Dammers, Diederik W. J. Dippel, Clemens M. F. Dirven, Mathieu van der Jagt, Fop van Kooten, Aad van der Lugt, Walter M. van den Bergh, Bert A. Coert, Marcella C. Müller, W. Peter Vandertop, Guus N. Beute, Annemarie W. Oldenbeuving, Bram van der Pol, Gerwin Roks, Willem Jan J. van Rooij, Menno Sluzewski

**Affiliations:** 1 Department of Intensive Care Medicine, UMC Utrecht Brain Center, University Medical Center Utrecht, Utrecht University, Utrecht, The Netherlands; 2 Department of Neurology and Neurosurgery, UMC Utrecht Brain Center, University Medical Center Utrecht, Utrecht University, Utrecht, 3508 The Netherlands; 3 Department of Intensive Care Adults and Erasmus MC Stroke Center, Erasmus MC — University Medical Center Rotterdam, Rotterdam, The Netherlands; 4 Department of Critical Care, University Medical Center Groningen, University of Groningen, Groningen, The Netherlands; 5 Department of Radiology, UMC Utrecht Brain Center, University Medical Center Utrecht, Utrecht University, Utrecht, The Netherlands

**Correction: Pilot Feasibility Stud 8, 193 (2022)**

10.1186/s40814-022-01155-4

Following publication of the original article [1], the authors reported an error in the Institutional Author group “HIMALAIA Study Group“, wherein affiliation 6 “University Medical Center Utrecht, Utrecht University Utrecht, The Netherlands” was linked to the HIMALAIA Study Group. Affiliation 6 should not be linked to the HIMALAIA Study Group.

The Institutional Author group has been updated above and the original article [1] has been updated.
